# Association Between Food Insecurity and Mental Health Among College Students in the Bronx, New York (NY)

**DOI:** 10.3390/nu17213485

**Published:** 2025-11-06

**Authors:** Collette M. Brown, Peter C. Nwakeze, Aditi Puri, Chesley Sanchez, Latoya Callender, Emily V. Williams, William Suarez

**Affiliations:** 1 School of Health Sciences, Human Services and Nursing, Lehman College, The City University of New York, Bronx, NY 10468, USA; latoya.callender11@login.cuny.edu (L.C.); william.suareziigomez@lehman.cuny.edu (W.S.); 2 School of Allied Health Professions, King Graduate School, Monroe University, Bronx, NY 10468, USA; pnwakeze@monroeu.edu (P.C.N.); apuri@monroeu.edu (A.P.); 3 Department of Epidemiology and Biostatistics, School of Public Health, SUNY Downstate Health Science University, Brooklyn, NY 11203, USA; chesley.sanchez@downstate.edu; 4 College of Arts and Sciences, Health Studies, American University, Washington, DC 20016, USA; emilyvwilliams452@gmail.com

**Keywords:** food insecurity, mental health, college students

## Abstract

**Background/Objectives**: Food insecurity and mental health are two important issues affecting college students, and their incidence have increased since the coronavirus disease (COVID-19) pandemic. The objective of this study was to examine the association between food insecurity and the mental health outcomes among college students in the Bronx, NY. **Methods**: The study utilized a cross-sectional design. Data were collected from 710 undergraduate students, using a self-administered survey with a quick response (QR) code. The survey consisted of sociodemographic questions, the US Household Food Security Survey Module (ten-item questionnaire), and the Depression, Anxiety, and Stress Scale (DASS-21). Data were analyzed using Statistical Package for the Social Sciences (SPSS), Version 29. **Results**: Results of the study indicated that 53% of the participants were food insecure, 47.4% had high depressive symptoms, 46.2% had high anxiety, and 50.6% had high stress levels. Multiple logistic regression revealed the reciprocal association between food insecurity and mental health. Students who were stressed (*p* = 0.022) were likely to experience food insecurity, while those who were food insecure were more likely to experience stress (*p* = 0.007) and depression (*p* < 0.021). Students who identified as Black (*p* = 0.021) and had lower income (*p* = 0.031) were more likely to be food insecure. **Conclusions**: This research shows that food insecurity worsens mental health, and mental health worsens food insecurity. However, it was not possible for us to establish causality between the two variables.

## 1. Introduction

The U.S. Department of Agriculture (USDA) [1] defines food insecurity as the unknown or limited availability of nutritional and safe foods, or the unknown or limited ability to obtain acceptable foods in socially acceptable ways. In 2023, 13.5% of U.S. households were considered food insecure at some point, with food insecurity statistically significantly higher than the 12.8% of U.S. households in 2022 [1]. These households, among many others with limited access to food, are often left with minimal food choices, resulting in poor dietary habits such as skipping meals or consuming a nutrient-poor diet [2,3,4,5,6], which have further implications for poor overall health. College students are a population significantly affected by food insecurity, often experiencing higher prevalence rates than their surrounding communities due to compounded challenges such as limited income, academic pressures, and insufficient access to affordable food [7,8,9]. In 2020, approximately 23% of university students in the U.S. were considered food insecure, with most of those students falling into a “very low food security range, meaning they reported multiple instances of eating less than they should or skipping meals because they could not afford enough food” [10]. Additionally, 59% of them who were potentially eligible for SNAP benefits were not receiving them. While this percentage is higher than regular US households, numerous studies have indicated prevalence between 20% and over 50% [11,12,13,14,15,16]. This further exacerbates the challenges of food insecurity for young adults, specifically in communities of color, low-income individuals, or other underserved communities [17,18,19].

Risk factors associated with food insecurity among college students include increased cost of education, being first-generation college students, high cost of living, being part of a low-income household, and being from a minority group [8,20,21,22]. For example, [22] conducted a longitudinal study over five years and found that only 47% of food-insecure first-generation students graduated, compared with 59% of their food-secure counterparts. In contrast, 65% of food-insecure non-first-generation students and 76% of food-secure non-first-generation students graduated, highlighting the intersecting challenges faced by first-generation students. In another study, [22] found that overall food insecurity among college and university students was 24%. Out of these food insecure populations, there were stark disparities between non-Hispanic Black populations (43%), transgender/non-binary populations (42%), and first-generation populations (33%).

Food insecurity is recognized as a psychosocial stressor that negatively impacts mental health, contributing to higher rates of psychological disorders such as stress, anxiety, and depression [15,23,24,25,26,27,28,29,30,31,32]. The World Health Organization [33] defines mental health as a state of well-being in which individuals can cope with stress, utilize their abilities effectively, succeed in learning and work, and engage actively in their community. In a recent review of the literature on food insecurity and psychological distress, [27] found that multiple studies over the last decade point to increased severity of depressive symptoms among college students who also reported being food insecure. In a multi-institutional study on food insecurity across US college campuses, [34] found that food-insecure students were more likely to report high stress compared to food-secure students.

Nutrition plays a vital role in both physical and mental well-being. Certain micronutrient deficiencies have been linked to the development of mental illnesses [35,36]. For example, deficiencies in vitamin B_12_ and iron can cause irritability, fatigue, depression, memory loss, and cognitive impairment, which are common symptoms of anxiety and depression [37]. Without the fundamental need of food for nourishment, stress and other mental health concerns are bound to arise [18]. This emotional strain stems from not having sufficient food, concerns about the quality of food, and having to choose between buying food and meeting another basic need. When individuals lack proper nutrition, they may feel significantly more stressed in situations that would otherwise be only mildly stressful. This effect can be even more pronounced in college students, who often face stress related to academic pressures, financial challenges, social expectations, and balancing other responsibilities. The lack of proper nutrition and stress can trigger both depression and anxiety. According to [38], not only is there an association between food insecurity and depression but also a strong correlation with depression becoming more severe as food insecurity worsens.

Aside from psychological stressors, food insecurity compounds other problems students face. Researchers [34] also reported that food-insecure students were at greater odds of having poor sleep quality, disordered eating behaviors, and a GPA of <3.0 compared to food-secure individuals. Oh et al. [23] found out that food insecurity was associated with perceived need for help, loneliness, and self-injurious behaviors among college students. Food security status is a major determinant of academic success. It significantly impacts grades and college dropout rates, particularly for first-generation college students [21].

Mental illness can impact a person’s ability to access safe and nutritious foods. A few studies have explored the impact of mental illness (severe mental illness and depression) on food insecurity [39,40,41,42,43]. In the study conducted by [39], participants with a two-year risk of depressive symptoms were approximately four times more likely to experience persistent food insecurity, especially in households with children. In another study [40], maternal depression had 1.5–2.03 times the odds of being associated with food insecurity in households with young children. In their study, most of these women (86%) were received WIC and approximately 40% received SNAP. In a systematic review conducted by [42], the authors reported that 40% of people with SMI were 2.71 times more likely to experience food insecurity compared to the general population. According to the National Alliance on Mental Illness [43], the presence of a serious mental illness (SMI) can hinder one’s ability to obtain healthy food, which contributes to food insecurity. While these studies examined the effects of mental health on food insecurity, none of them focused on college students.

A growing body of research has emphasized the bidirectionality of food insecurity and mental health [44,45,46,47], suggesting that each condition exacerbates the other in a cyclical manner. In an integrative review of the bidirectional relationship between food insecurity and mental health among Canadian immigrants, [46] indicated that these variables were intricately linked and were mediated by factors such as comorbidities, poverty, and societal stigma. Additionally, [45] also found that significant bidirectional relationships were observed between food insecurity and psychological distress in a prospective study among cigarette smokers. 

The New York State Health Department [48] indicates that the Bronx has the highest prevalence of food insecurity among adults (39%) compared to other counties in the state. This population is uniquely impacted by socioeconomic and structural challenges disproportionate to other regions across the country. College students are vulnerable to both food insecurity and mental health issues, often experiencing these challenges simultaneously. Although many studies were conducted on food insecurity and mental health independently among college students, none has focused on how these two variables affect each other. Given that both food insecurity and mental health can affect individuals simultaneously, it is important to investigate their interconnections, especially among college students in an urban environment. To our knowledge, no studies have simultaneously shown how food insecurity affects mental health and how mental health affects food insecurity among college students. Therefore, this study examined the association between food insecurity and the mental health and vice versa among college students in the Bronx, NY. We hypothesized that there is an association between food insecurity and mental health, where students who experience food insecurity are more likely to report mental health conditions, and those who experience mental health conditions are also likely to experience food insecurity.

## 2. Materials and Methods

### 2.1. Study Design and Setting

The study employed a quantitative, cross-sectional design to examine the bidirectional association between food security status and mental health (depression, anxiety, and stress) among undergraduate students in the Bronx, NY, USA. The sampling procedure utilized a convenience sample of students enrolled in private and public colleges due to the ease of access of the participants to the researchers.

### 2.2. Participants and Recruitment

Seven hundred and ten students participated in the study. Eligibility criteria for participation included being an undergraduate student aged 18 years or older. Researchers obtained the necessary permissions to access the classrooms before data collection.

### 2.3. Data Collection Procedure

The electronic survey was displayed via a QR code. The survey included an informed consent form that students were required to read. At the end of the informed consent form, students were asked, “Do you want to participate in this study?” Affirmative consent was obtained when students selected “yes.” Students were also asked to take a screenshot of the informed consent for their records. Students then completed the self-administered questionnaire. Data were collected from January to May 2024.

### 2.4. Measures

#### 2.4.1. Food Security Scale:

Food insecurity (FI) was assessed using the ten-item US Household Food Security Survey Module questionnaire developed by the US Department of Agriculture, Economic Research Service [1]. The instrument has a Cronbach alpha of 0.856 [49]. The survey contained questions about the availability of food eaten in participants households in the last 12 months, and whether they were able to afford the food that they needed. Participants responded to statements such as “In the last 12 months, did you ever eat less than you felt you should because there wasn’t enough money for food?” and “In the last 12 months, were you every hungry but didn’t eat because there wasn’t enough money for food?” Responses to these questions were *yes, no,* and *don’t know*. Other questions included “The food that (I/we) bought just didn’t last, and (I/we) didn’t have money to get more.” Responses were *often, sometimes, or never true* for (you/your household) in the last 12 months.

#### 2.4.2. Mental Health Status

The Depression, Anxiety, and Stress Scale (DASS-21) was used to evaluate the mental health status of the participants [50]. The DASS-21 consists of three subscales, each consisting of 7 statements to measure depression, anxiety, and stress. The internal reliability of the total DASS-21 scale is 0.93. The reliability for the depression, anxiety, and stress subscales is 0.88, 0.82, and 0.90, respectively [51]. Students were asked to indicate on a scale of 0–3 how frequent each statement applied to them in the past week. Sample statements from each the depression, anxiety, and stress subscales include “I found it difficult to work up the initiative to do things,” “I was worried about situations in which I might panic and make a fool of myself,” and “I found it difficult to relax”, respectively.

#### 2.4.3. Covariates

Data on sociodemographic factors such as age, gender, race, income, and educational level were collected. Data were also collected on whether college campuses had a food pantry. Responses were *Yes = 1, No = 0,* and *I don’t know = 0*. Students were asked if they received food from the pantry, and whether they received SNAP or Women, Infant, and Children (WIC) supplements. Responses were dichotomized as *Yes = 1* or *No = 0*. Students were also asked to indicate their Grade Point Average (GPA). We further categorized the responses for GPA into <2.5, 2.5–3.49, and 3.5–4.0.

### 2.5. Data Analysis

Data were analyzed using the Statistical Package for the Social Sciences (SPSS), version 29 [52]. Descriptive statistics were presented in counts and percentages, followed by Chi-square test to assess bivariate associations. A multiple logistic regression model was then used to statistically control for variables that might distort the overall findings.

We created a food insecurity index using the 10-item US Household Food Security Survey. Each item response was coded as “affirmative = 1” or “non-affirmative = 0” following US Department of Agriculture guidelines. Regarding items with response categories “often,” “sometimes,” “never”), “often” or “sometimes” were coded as affirmative; “don’t know” responses were initialized as a system missing value. For items with “yes” or “no” responses, “yes” was coded as affirmative, and “no” as negative or non-affirmative. Further, for items with responses in the form of counts or frequencies (e.g., “almost every month”, “some months but not every month,” “only one or two months”), the last category was coded as affirmative. We created the food insecurity index by summing the affirmative responses. Scores between 0 and 1 indicated high or marginal food security; scores between 2 and 4 indicated low food security, and scores between 5 and 6 indicated very low food security. The responses were dichotomized into food security (high or marginal food security) and food insecurity (low or very low food security).

For the DASS-21 instrument, each subscale was summed and multiplied by 2 to determine the levels of depression, anxiety, and stress they experienced. Depression, anxiety, and stress subscale scores were categorized into *normal* (0–9, 0–7, 0–14), *mild* (10–13, 8–9, 15–18), *moderate* (14–20, 10–14, 19–25), *severe* (21–27, 15–19, 26–33), or *extremely severe* (28+, 20+, 34+), respectively. Depression, anxiety, and stress variables were further recoded into and high categories to facilitate Chi-square and regression analyses.

To explore the association between food insecurity and mental health (specifically stress, anxiety, and depression), we treated food insecurity and mental health both as dependent and independent variables in four logistic regressions. Mental health variables were recoded into binary forms: stress, anxiety, and depression were recoded as (0 = normal/mild, 1 = moderate/severe) and food insecurity was also recoded as (0 = food insecure, 1 = food secure). The models incorporated specific covariates, including age, ethnicity, education, income, participation in SNAP or WIC programs, and the presence of children in the household.

To evaluate the strength and explanatory power of these models, we conducted a comparative analysis using log-odds (lnExp(B) and Nagelkerke R^2^ values. Log-odds were used instead of odds ratios to facilitate direct comparison of effect direction and magnitude across models [53,54]. *p*-values of <0.05 were considered statistically significant.

### 2.6. Ethical Consideration

The study was approved by the Institutional Review Boards of the colleges involved in the study (IRB No. 2024-0087, Lehman College and Monroe College (now Monroe University) IRB No: FAC-2023-04). Students read the informed consent form attached to the beginning of the electronic survey. If they agreed to participate after reading the informed consent form, they would respond “yes” to the question “Do you want to participate in this study.” The questions were displayed to them after the affirmative.

## 3. Results

### 3.1. Demographic Characteristics of the Participants

A total of 710 students participated in the study. As shown in Table 1, the sample is mostly young, with 67.1% aged 18–24, suggesting that the population consists mainly of traditional college-aged students. Regarding the educational breakdown, first-year students account for close to 40% of respondents. Females make up 74.3%**,** nearly tripling the percentage of their counterparts. There is notable racial and ethnic diversity. The largest groups are Black/African American (44.4%) and Hispanic (41.5%)**,** showing that the study captures experiences from under-represented students. Whites (6%) and minority groups like Native Hawaiian/Pacific Islander (0.7% and 1.4%, respectively), are under-represented. The Table further shows that 56.4% earn below USD 40 K, suggesting that greater proportions are low-income students. Ony 29.8% reported household incomes exceeding USD 40 K. GPA distribution shows 60.3% reporting between 2.5 and 3.49 and only 9.9% under 2.5, indicating a strong academic performance.

Regarding food insecurity and resource use, 53% of participants report food insecurity, an interesting finding that underscores the need for food assistance on campuses. Despite this, only 34.3% were aware of the existence of food pantries on the campuses, and close to 49% said they “don’t know.” Among those aware, less than half ever used the service.

Results of the study indicated that participants experienced overwhelming psychological challenges. Approximately 47% of the participants had high depressive symptoms, 46% had high anxiety, and 51% experienced high stress levels.

### 3.2. Demographic Variables, Psychological Factors and Food Security Status

Table 2, Table 3, Table 4 and Table 5 display the bidirectional association between food insecurity and mental health outcomes (stress, anxiety, and depression) among undergraduate college students. Two sets of logistic regression models were conducted. The first regression analysis (Table 2) focused on the effect of each mental health variable on food insecurity. Regression analyses for Table 3, Table 4 and Table 5 examined how food insecurity predicted each mental health variable. For each model, we reported the odds ratios, 95% confidence intervals, and Pseudo R-Square values. We used them to compare precision and strength of association across the models. The Pseudo R-Square provided us with insight into the relative fit of the models.

Table 2 shows the effect of mental health on food insecurity, controlling for age, race/ethnicity, income, SNAP participation, and presence of children. Stress (*p* = 0.022, Exp(B) = 0.518) and depression (*p* = 0.054; Exp(B) = 0.518) were predictors of food insecurity, but not anxiety (*p* = 0.233; Exp(B) = 1.506). Students who have higher stress and depression are more likely to be food insecure. Also, students who are Black/African and had lower income were more food insecure.

Table 3 presents the effect of food insecurity on stress, controlling for age, race/ethnicity, income, SNAP participation, and presence of children. Only food insecurity and having children were significantly associated with stress. Students who were food secure reported lower stress levels compared to those experiencing food insecurity (*p* = 0.007, Exp(B) = 0.495). Additionally, students living with children reported less stress than those without children.

Table 4 shows the results for anxiety. Students aged 25–34 were more likely to experience anxiety than those aged 35 or older (*p* = 0.024, Exp(B) = 2.840). However, food insecurity itself was not significantly associated with anxiety (*p* = 0.430, Exp(B) = 0.810).

Table 5 reports the effect of food insecurity on depression. Here, food insecurity was the only variable significantly associated with depression (*p* = 0.021, Exp(B) = 0.523). Students who were food secure had lower depression. No other variable was significant.

When food insecurity was modeled as the predictor, ln(OR) values were −0.703 for stress, −0.211 for anxiety, and −0.648 for depression, with corresponding Nagelkerke R^2^ values of 0.071, 0.076, and 0.119 (Table 6). When mental health variables were modeled as predictors of food insecurity, ln(OR) values were −0.658 (stress), +0.410 (anxiety), and −0.662 (depression), each with an R^2^ of 0.110. We also calculated the average absolute ln(OR) and mean Nagelkerke R^2^ to assess overall impact. Mental health variables demonstrated slightly stronger predictive power for food insecurity (average ln(OR) = 0.577 vs. 0.521; mean R^2^ = 0.110 vs. 0.089).

## 4. Discussion

The present study examined the bidirectional relationship between food insecurity and mental health conditions (stress, anxiety, and depression) among college students in the Bronx, NY. These findings suggest a possible reciprocal association between food insecurity and mental health, which would be best tested in longitudinal data. This demonstrates the importance of addressing both material hardships, typically the focus of public health and nutrition interventions, and psychological distress, which is often managed by mental health professionals such as social workers, psychologists, and psychiatrists.

Results of the study indicated that participants experienced overwhelming psychological challenges. Approximately 47% of the participants had high depressive symptoms, 46% had high anxiety, and 51% experienced high stress levels. These results are consistent with previous studies [12,20]. These high levels of psychological distress could be the result of financial problems, challenges navigating school full-time, being food insecure, and being deprived of the necessities of life. These levels of psychological distress could also result from cumulative effects of the COVID-19 pandemic.

Our results indicate that students who have higher stress are more likely to be food insecure. Additionally, those who were depressed were more likely to be food insecure, however the association was only marginally significant. Our findings are consistent with previous studies [39,40,41,42,43]. In three of the studies [39,40,42], participants who were depressed were 2–4 times more likely to experience food insecurity. Depression and stress can be associated with reduced school and work performance, job loss, impaired decision-making [55,56], loss of interest in usually enjoyable activities, and distorted thinking. Individuals experiencing these challenges with depression and stress may struggle to purchase, plan, and eat nutritious foods. Individuals may opt for foods that are convenient and readily available, which are often lacking in nutritional value. In some instances, they may neglect to ensure adequate food intake or fail to maintain a regular eating schedule, leading to skipped meals. These cumulative effects may contribute to experiencing food insecurity.

Our study also indicates that food insecurity predicted increased mental health outcomes (stress and depression), but not anxiety. In Table 3, food insecurity and having children are significantly associated with stress. Students who were food secure reported lower stress levels compared to those experiencing food insecurity. However, students living with children reported less stress than those without children. This finding is inconsistent with previous studies [57,58]. However, it is reported that while parents may experience high stress when they have up to three children, having four or more brings less stress [59]. In our study, we did not analyze the number of children that students had. It is plausible that parenting students have learned better time management and self-regulation than non-parenting students. It is also possible that parenting students have robust social support systems, especially from their parents and grandparents in managing childcare demands.

Similarly, in Table 4, food insecurity is associated with depression. A recent study by [60] reported that emergent food-secure students had higher odds of anxiety and depression (OR range 2.35–2.85) than those who had persistent food insecurity. Although our study did not categorize participants by the duration of food insecurity, the findings nonetheless provide evidence of a significant association between food insecurity and depression. Other studies reported similar associations between food insecurity and depression [14,23,24,25,27,28]. When students face both food insecurity and psychological distress, the combined impact can significantly disrupt their sleep, hinder their class attendance, impair their concentration, and ultimately jeopardize their academic success, even leading to college dropout.

Although the prevalence of food insecurity among the general population in the Bronx was 39% [48], our sample of college students indicate a significantly higher prevalence. Regarding food insecurity, 53% of participants reported food insecurity. Our results are consistent with previous studies [7,8,9,11,12,13,14,15,16] that reported a higher prevalence among college students than the general population. Given that the Bronx has the highest food insecurity rate among the 62 counties in the state of New York, and more than half of the college students sampled in the county are food insecure, expedited interventions are needed to ameliorate the problem. Food insecurity has increased since COVID-19 and has impacted students’ educational trajectory. Students who are food insecure are more likely to miss classes, withdraw from school, have lower grades because they cannot concentrate, and may eventually delay their graduation. This finding suggests the need for food assistant programs on all college campuses.

The sociodemographic factors of race and income were associated with food insecurity in our study. African American students were more likely to be food insecure compared to white students. Similarly, students with higher income were more food secure than those earning a lower income. Similar results were documented in previous studies [14,61,62]. Students from higher-income households are generally more food secure because they have the finances to purchase safe and nutritious foods and are less likely to worry about paying for tuition, housing, or other expenses. They are also less likely to ration their food or experience hunger.

Approximately 34% of students were aware that their college has a food pantry and less than one-half of them utilized this resource. College food pantry is a great source of food for students who need supplemental nutrition. Students in a California study reported that some of their reasons for utilizing the college food pantry were that they ran out of food and were worried to spend money to buy more, did not want to run out of food, they ran out of food but did not have the time to buy food/groceries, or they used the food pantry to supplement their nutritional needs because they did not have enough [13]. Not all colleges have this resource, but for those who do, having a food pantry on campus can serve to reduce food insecurity [63,64] and alleviate the stress and anxiety associated with food insecurity, especially at a time when students are faced with increased financial constraints. Currently, the price of rent, food, transportation, and tuition are increasing, while the job market and wages have not kept up to pace with inflation [65]. The underutilization of the food pantries could be due to stigma, as many students do not want their peers to see them accessing foods from the pantry.

Our study revealed that approximately 56% of students who received SNAP were food insecure compared to 46% who did not receive SNAP. This indicates that even when students received SNAP, they still report a high prevalence of food insecurity. Previous research conducted by [9] reported a similar finding. They reported that students who were receiving SNAP and those with lower monthly discretionary budgets were more likely to be food insecure. This suggests that SNAP recipients might be financially vulnerable, even with assistance from the government. In our study, approximately 74% of the students reported a household income of less than or equal to USD 60,000. Living in a metropolitan area like New York City and earning a household income of less than USD 60,000 is not financially adequate, given the continued increase in the cost of living, especially for housing and food. These realities further highlight the difficulties students face in providing the basic needs to survive, attend college, and maintain a GPA that is enough to qualify for graduation.

SNAP is the largest food assistance program that is funded by the federal government to assist low-income families to supplement their grocery budget [66]. Receiving SNAP should help to alleviate food security and subsequent negative health outcomes associated with it. However, students might not participate in SNAP for various reasons. Some students may not know if they qualify for SNAP, while others may find it difficult to enroll in SNAP due to the eligibility criteria set by the government. To qualify for SNAP, students must be enrolled at least half-time in an institution of higher education and meet other criteria, such as working at least 20 h per week, caring for a child, unable to work, participating in a work–study program, or receiving public assistance, among others [67]. These complex criteria and sometimes confusing language leave students unsure of their eligibility status, and they might not pursue completing the form [11]. In other cases where students obtain help to complete the form, they might be denied if forms are not completed correctly [11]. In addition, students may not participate in SNAP because they do not want to experience stigma associated with using the card. In a study conducted by [68], 56% of SNAP recipients reported that they experienced stigma, and of those, most of them were younger individuals. These challenges pose a greater risk of being food insecure.

There are several limitations to this study. This study utilized a cross-sectional study design and convenience sampling method; therefore, we were unable to make causal inferences between food insecurity and mental health. Since the participants represented only two universities in the Bronx and a convenience sampling was used, our findings are not representative of the universities in the Bronx, and generalizations cannot be made beyond the study participants. We did not conduct a formal a priori power to ensure that the study is statistically capable of detecting meaningful effects. The study is subjected to recall bias because questions on food security ask students to recall the food status within the past year. They may have difficulty remembering their food accessibility and consumption patterns, which might lead to providing inaccurate information. While we know that there are mental health services provided by the campuses, we were unable to determine if the sampled students were able to access this service. Finally, students self-reported their GPA; however, we were unable to verify the accuracy of the data. This analysis suggests bidirectional associations between the two variables using reciprocal logistic models. The results are consistent with the syndemic theory. Longitudinal studies are needed to confirm bidirectional association between food insecurity and mental health outcomes.

## 5. Implications

There is a bidirectional relationship between food security and psychological health [47]. Moreover, college students who experience both are likely to have greater negative health and academic impacts. They may consume unhealthy diets (eat more calorie-dense foods). When students do not know where their next meal is coming from, ration their foods and stay hungry, or do not have the finances to purchase healthy and nutritious foods, additional stress, anxiety, and depression are created. These psychological effects might be compounded by their household composition, especially if they have children. Poor nutrition, psychological problems, and taking care of children can lead to poor health outcomes, which can have a direct impact on their academic performance. A study conducted by [9] indicated that food insecurity was significantly associated with psychosocial health and that psychosocial health was associated with GPA. In a systematic review conducted by [69], they mentioned that Hispanic and Black mothers were almost twice and more than twice at risk of experiencing depression, respectively, compared to their white counterparts. Also, when households are food insecure, this impacts everyone, causing them to consume unhealthy foods and not providing balanced nutrients to their diets. Therefore, the burdens of food insecurity and psychological challenges contribute to many negative aspects of a person’s life.

Food insecurity puts college students at an elevated risk for stress, anxiety, and depression, which can compound the negative academic and health consequences that they face. Research consistently demonstrates that food-insecure students report significantly higher levels of perceived stress and depressive symptoms compared to their food-secure peers [27,70,71]. Research also suggests that persistent or emergent food insecurity predicts increased odds of depression and anxiety over time, as well as disrupted sleep patterns [60]. These psychological burdens can reduce students’ ability to attend classes, concentrate, complete assignments, and maintain a satisfactory GPA, thereby jeopardizing retention and graduation rates [16,70]. In addition, if students have high levels of psychological problems, they will be less motivated to seek job opportunities or keep their jobs, which will further increase their food insecurity levels. Therefore, addressing food insecurity and providing access to mental health services are crucial for college students, especially students who live in low-income households and may not have the finances to purchase nutritious foods or afford mental health services off campus.

## 6. Recommendations

The persistent and increased incidence of food insecurity and mental health challenges among college students warrants immediate and multifaceted programs and policy intervention to address these problems simultaneously. This includes offering a holistic suite of services, including counseling, academic advising, financial aid guidance, and referrals to community resources, to address the multifaceted needs of food-insecure and mentally distressed students.

Firstly, providing awareness of food pantry and mental health services are very important. In our study, approximately 49% of the students reported that they were aware that their college has a food pantry. Advertising such resources through campus media, physical signage in high-traffic areas on campus, and classroom visits may provide awareness about these services. Secondly, students who may qualify for SNAP may need assistance to complete the form. Recognizing the nuances in completing the SNAP application, we recommend that college campuses provide resources to help students to complete the application and expand SNAP eligibility. Thirdly, since our study indicated that there is a bidirectional association between mental health and food insecurity, we recommend that colleges create an integrated approach that assists students with food and counseling services for those in need. Even though we have performed this analysis, we cannot establish causality because of lack of temporality of occurrence of the key variables. This can only be achieved through conducting a longitudinal study. Colleges can also advocate to limit the restrictions of SNAP for college students. At the individual level, colleges should promote workshops for students that focus of stress-reduction and mindfulness training to help students cope with mental health issues. Also, encouraging students to form social groups may help to minimize the effects of stress, anxiety, and depression.

## 7. Conclusions

It is well established in the literature that there is an association between food insecurity and psychological distress (stress, anxiety, depression) among college students. This research shows that food insecurity worsens mental health, and mental health worsens food insecurity. However, it was not possible for us to establish causality between the two variables. More specifically, more than half of the sampled students were food insecure and close to half of them had severe to extremely (high) psychological issues. Students who use SNAP were more food insecure than those who did not use SNAP, highlighting a greater problem than just food insecurity. While college campuses are trying to mitigate the effects on students by providing resources, it is unclear why more students are not accessing the services. This research provides useful insights for college campuses to provide interventions to benefit students who are food insecure and/or have psychological issues, ultimately enhancing their capacity to focus on their academic trajectory.

## Figures and Tables

**Table 1:** Demographic characteristics of participants **^n^**.

Variables	Categories	Numbers	Percentages
Age (n = 709)	18–24	476	67.1
	25–34	141	19.9
	>34	92	13.0
Gender (n = 704)	Male	181	25.7
	Female	523	74.3
Race/Ethnicity (n = 703)	AI/AN (non-Hispanic)	10	1.4
	Asian (non-Hispanic)	42	6.0
	Black/African American	312	44.4
	Hispanic	292	41.5
	Native Hawaiian/Pacific Islander (non-Hispanic)	5	0.7
	White	42	6.0
Education (n = 710)	Freshmen	280	39.4
	Sophomore	147	20.7
	Junior	136	19.2
	Senior	147	20.7
Income (n = 709)	<20 K	211	29.8
	20,000–40,000	191	26.9
	40,001–60,000	127	17.9
	60,001–80,000	80	11.3
	>80,000	80	11.3
	Missing	20	2.8
GPA (n = 657)	<2.50	65	9.9
	2.50–2.3.49	396	60.3
	3.50–4.0	196	29.8
Food Pantry (n = 706)	Yes	248	34.3
	No	107	14.8
	Do not Know	351	48.6
Received Food from Pantry (n = 247)	Yes	104	41.9
	No	143	57.7
Received SNAP (n = 705)	Yes	133	18.9
	No	572	81.1
Received WIC (n = 706)	Yes	45	6.4
	No	661	93.6
Have Children (n = 199)	Yes	53	26.7
	No	146	73.3
Food Security (n = 710)	Food Secure	334	47.0
	Food Insecure	376	53.0
Depression (n = 709)	Low	173	24.4
	Medium	200	28.2
	High	336	47.4
Anxiety (n = 707)	Low	172	24.3
	Medium	209	29.5
	High	326	46.2
Stress (n = 708)	Low	199	28.1
	Medium	151	21.3
	High	358	50.6

^n^ = Sample sizes varied across variables due to item nonresponses.

**Table 2:** Logistic regression of mental health on food insecurity.

Mental Health on Food Insecurity							
	B	S.E.	Wald	df	Sig.	Exp(B)	95% CI for Exp(B)
Age 18–24	−0.166	0.478	0.121	1	0.728	0.847	0.332–2.163
Age 25–34	−0.421	0.452	0.869	1	0.351	0.656	0.271–1.591
American Indian/Alaskan Native/Non-Hispanic	−0.819	0.966	0.718	1	0.397	0.441	0.066–2.930
Asian non-Hispanic	−1.217	0.722	2.838	1	0.092	0.296	0.072–1.220
Black/African American Non-Hispanic	−1.392	0.602	5.352	1	0.021	0.249	0.076–0.808
Hispanic	−1.118	0.606	3.410	1	0.065	0.327	0.100–1.071
Native Hawaiian Other Pacific	−1.137	1.534	0.550	1	0.458	0.321	0.016–6.483
Income	0.850	0.394	4.659	1	0.031	2.340	1.081–5.066
Received SNAP	−0.331	0.252	1.732	1	0.188	0.718	0.438–1.176
Have Children	0.469	0.417	1.265	1	0.261	1.598	0.706–3.615
Stress	−0.657	0.288	5.211	1	0.022	0.518	0.295–0.911
Anxiety	0.410	0.344	1.421	1	0.233	1.506	0.768–2.953
Depression	−0.662	0.344	3.711	1	0.054	0.516	0.263–1.012
Constant	1.607	0.966	2.767	1	0.096	4.990	

**Table 3:** Logistic regression of food insecurity on stress.

	Food Insecurity on Stress						
	B	S.E.	Wald	df	Sig.	Exp(B)	95% CI for Exp(B)
Food insecurity	−0.703	0.260	7.319	1	0.007	0.495	0.297–0.824
Age 18–24	−0.231	0.520	0.198	1	0.657	0.793	0.286–2.200
Age 25–34	0.352	0.476	0.546	1	0.460	1.421	0.559–3.614
American Indian/Alaskan Native/Non-Hispanic	0.826	1.232	0.450	1	0.502	2.285	0.204–25.556
Asian non-Hispanic	−0.204	0.727	0.079	1	0.779	0.815	0.196–3.388
Black/African American Non-Hispanic	−0.243	0.574	0.179	1	0.672	0.784	0.255–2.415
Hispanic	−0.097	0.577	0.029	1	0.866	0.907	0.293–2.813
Income	0.653	0.454	2.069	1	0.150	1.921	0.789–4.575
Received SNAP	−0.126	0.273	0.213	1	0.644	0.881	0.516–1.506
Have Children	0.912	0.458	3.971	1	0.046	2.490	1.015–6.106
Constant	0.083	0.0977	0.007	1	0.932	1.086	

**Table 4:** Logistic regression of food insecurity on anxiety.

	Food Insecurity on Anxiety						
	B	S.E.	Wald	df	Sig.	Exp(B)	95% CI for Exp(B)
Food insecurity	−0.210	0.267	0.623	1	0.430	0.810	0.480–1.366
Age 18–24	0.771	0.494	2.436	1	0.119	2.163	0.821–5.698
Age 25–34	1.044	0.462	5.113	1	0.024	2.840	1.149–7.020
American Indian/Alaskan Native/Non-Hispanic	−0.173	1.066	0.026	1	0.871	0.841	0.104–6.803
Asian non-Hispanic	−0.671	0.744	0.812	1	0.367	0.511	0.119–2.199
Black/African American Non-Hispanic	−0.184	0.619	0.088	1	0.766	0.832	0.247–2.798
Hispanic	−0.243	0.620	0.153	1	0.696	0.785	0.233–2.647
Income	0.015	0.426	0.001	1	0.971	1.015	0.441–2.339
Received SNAP	−0.246	0.280	0.772	1	0.379	0.782	0.451–1.354
Have Children	0.660	0.447	2.183	1	0.139	1.935	0.806–4.643
Constant	−0.096	1.004	0.009	1	0.924	0.908	

**Table 5:** Logistic regression of food insecurity on depression.

	Food Insecurity on Depression						
	B	S.E.	Wald	df	Sig.	Exp(B)	95% CI for Exp(B)
Food insecurity	−0.648	0.281	5.330	1	0.021	0.523	0.302–0.901
Age 18–24	0.844	0.500	2.855	1	0.091	2.326	0.874–6.192
Age 25–34	0.686	0.459	2.232	1	0.135	1.986	0.807–4.888
American Indian/Alaskan Native/Non-Hispanic	0.523	1.322	0.157	1	0.692	1.687	0.127–22.493
Asian non-Hispanic	−0.800	0.822	0.946	1	0.331	0.449	0.090–2.252
Black/African American Non-Hispanic	−0.321	0.692	0.216	1	0.642	0.725	0.187–2.812
Hispanic	−0.626	0.689	0.826	1	0.363	0.535	0.139–2.062
Income	0.527	0.490	1.156	1	0.282	1.693	0.648–4.422
Received SNAP	−0.412	0.289	2.028	1	0.154	0.663	0.376–1.168
Have Children	0.760	0.442	2.957	1	0.086	2.139	0.899–5.089
Constant	0.474	1.048	0.204	1	0.651	1.606	

**Table 6:** Establishing bidirectionality: comparing log-odds (ln()R) and Nagelkerke R^2^ across models.

Predictors	Exp(B)	95% CI	Ln(OR)	Nagelkerke R^2^
Food Insecurity on Stress	0.495	0.297–0824	−0.703	0.071
Food Insecurity on Anxiety	0.810	0.480–1.366	−0.211	0.076
Food Insecurity on Depression	0.523	0.302–0.907	−0.648	0.119
Food Insecurity on Mental Health Overall Impact (ln(OR): 0.521; Overall Variance explained by Nagelkerke: 0.089				
Stress on Food Insecurity	0.518	0.295–0.911	−0.658	0.094
Anxiety on Food Insecurity	1.506	0.768–2.953	0.410	0.069
Depression on Food Insecurity	0.516	0.253–1.012	−0.662	0.088
Mental Health on Food Insecurity Overall Impact (ln(OR): 0.577; Overall Variance explained by Nagelkerke: 0.084				

## Data Availability

The data presented in this study are available on request from the corresponding author. The data are not publicly available due to privacy and ethical restrictions.

## Abbreviations

The following abbreviations are used in this manuscript:

SNAP	Supplemental Nutrition Assistance Program
DASS-21	Depression, Anxiety, and Stress Scale
COVID-19	Coronavirus Disease 2019
FI	Food Insecurity
